# Food safety culture and the control of microbial communities in food production environments

**DOI:** 10.1038/s44259-025-00178-0

**Published:** 2026-01-27

**Authors:** Oleksii Omelchenko, Maria Diaz, Ana Victoria Gutiérrez, Mark A. Webber, Nicola Wilson, Matthew Gilmour

**Affiliations:** 1 Quadram Institute Biosciences, Norwich Research Park, Norwich, Norfolk, UK; 2 Norwich Medical School, University of East Anglia, Norwich, Norfolk, UK; 3 Centre for Microbial Interactions, Norwich Research Park, Norwich, Norfolk, UK; 4 Food Safety Research Network, Norwich Research Park, Norwich, Norfolk, UK

**Keywords:** Biotechnology, Microbiology

## Abstract

Food safety risks are controlled in agrifood settings by reducing the microbial burden in food ingredients and food production environments. Hygiene programmes involve an incremental implementation of chemical treatments (e.g., disinfectants) and engineering controls (e.g., elimination of susceptible harbourage sites). The strategies to disrupt the presence and transmission of microbial risks to foods are being refined by advanced microbiology and genomics that provide actionable evidence on the precise nature of local ecologies.

## A culture of food safety

With a commitment to the health and safety of their customers, food businesses implement a multitude of strategies to influence and reduce the risk of microbial contamination in the foods they produce. Even with the use of cleaning and disinfection agents, food production environments (FPEs) are not sterile. Resident microbiota are shaped by selective pressures, including temperature, exposure to disinfectants, and the continual introduction of new microbial species from the environment or from food ingredients^1^. Ultimately, adapted microbial communities of environmental, spoilage and pathogenic organisms can establish niches in FPE that can spill over to contaminate foods and food ingredients. As such, despite long-standing investment in hygiene, regulation, and testing, foodborne illness continues to represent a major public health burden, with not all potential sources identified. For example, in the United Kingdom, the UK Health Security Agency has recently reported continued increases in cases for each of Shiga toxin-producing *Escherichia coli* (STEC), *Listeria monocytogenes*, *Salmonella*, and *Campylobacter* ranging from 14% to 26%^2–5^. These trends highlight the pressing need for coordinated action from food producers, researchers, and the government to strengthen surveillance and develop more effective tools for control.

**Fig. 1:**
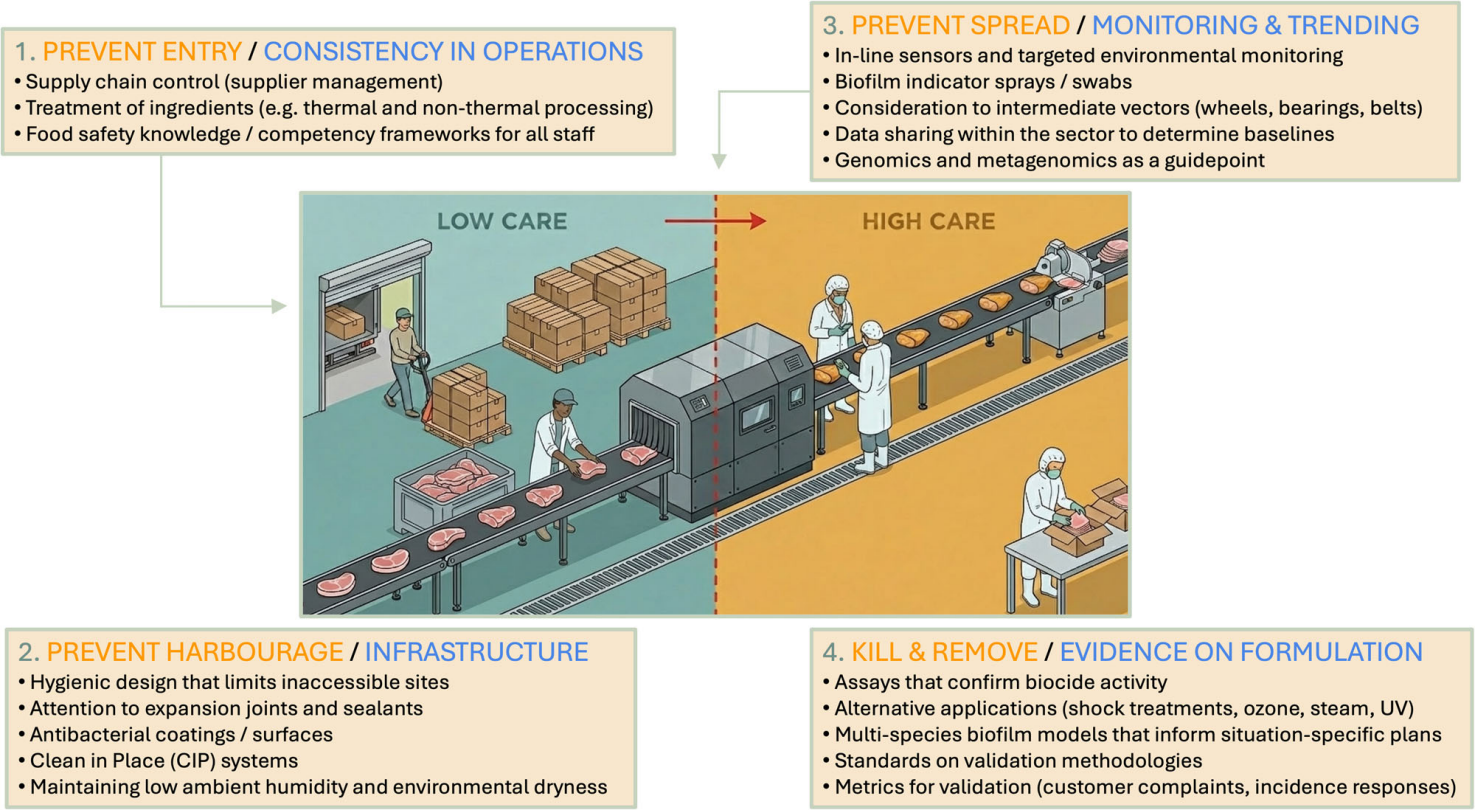
Opportunities for new scientific tools to implement and validate food safety control measures to mitigate microbial risks in industrial food production environments (FPE). Elements of the figure are discussed throughout this perspective. Control measures for microbial risks differ substantially between ‘low care’ and ‘high care’ zones. Low care areas, such as product storage zones, handle items unlikely to pose a contamination risk or that will undergo a kill step later. In contrast, high care areas manage ready-to-eat products or those with no further processing steps to reduce contamination risk. These areas may be physically connected, and all areas are subject to good manufacturing practices, including cleaning, zoning, and hygiene^15^. The central image (excluding the four surrounding text boxes) was created with a generative AI tool and adapted by the authors. Connected text boxes: Food Safety Control > Quality by Design Process > Solutions

Within the overall culture of food safety and processes of a food business^6,7^, the best defences against the presence and transmission of microbial risks are to prevent their entry, harbourage, and spread within FPE (Fig. 1). Commitment to food safety not only protects the health of consumers, but it also protects the reputation of brands. In many countries, it is the responsibility of each business to make safe food, and achievable frameworks for control of microbial contamination involve good hygiene practices and structured risk management plans such as the Hazard Analysis and Critical Control Point System (HACCP) that are customised to the specific circumstances of individual business^8–10^. Cleaning and disinfection (C&D) are a cornerstone of hygienic practice in food production^11,12^. Hygiene is further supported and iteratively improved within individual food businesses through the principles of ‘quality by design’^13,14^, whereby operations, infrastructure, and formulation of disinfectants are optimised using monitoring programmes that detect microbial risks and enable targeted and precise control measures amongst an overall backdrop of hygienic design. Guidance and standards are available to businesses from government and industry sources^15,16^ that further identify sector-specific or generalised control measures. These resources also support the development of training and certification programmes to support employee knowledge and to entrench the core principles and awareness of food safety across a business. As such, food safety culture is strengthened not only by monitoring and design, but also by supporting ongoing decision making through training, mentorship, and staff engagement at all operational levels. It is against these frameworks that we will provide a perspective on contemporary approaches and the scientific needs to develop biocontrol strategies that can result in demonstrable value to food safety and risk management.

In this perspective, we consider indoor FPEs that represent industrialised production settings for raw and ready-to-eat foods. We use language from microbial ecology^17^ to frame food safety strategies as being informed and optimised based on evidence of microbial **‘presence’** (at specific sites within a FPE), followed by **‘transmission’** (via exposure vectors), **‘connectivity’** (or, microbial **spread** between different components of a FPE), and **‘evolution’** (including microbial selection and adaptation at new sites). We use the term **‘resistance’** to refer to microbes that remain detectable following application of a biocide at a ‘during use’ concentration - that is, survival observed in real-world settings or during laboratory testing that replicates the effective concentration of an agent at a particular site^18,19^. Bland et al. further review terminology such as ‘resistance’ and ‘tolerance’, as applied in the food industry^20^. This perspective has been written largely from a view of UK practices, and we acknowledge that low- and middle-income countries (LMICs) face challenges, including infrastructural, economic, and regulatory constraints, that are outside the scope of this perspective.

We also refer the reader to the recent review on bacterial biocide susceptibility testing^21^ that presents the state of knowledge on biocides, that by virtue of their chemical nature, such as chlorine-based disinfectants, can act broadly against microbial cells; are used commonly and often at high concentrations; are meant to be fast-acting; and are used in a wide variety of settings, ranging from domestic, to animal and human health, to industrial settings. O’Reilly et al. also highlighted the challenges in standardising methods for assessing biocide activity^21^. These challenges arise, in part, because of the multitude of variables to consider, including biocide concentration, exposure time, state of the microbial test species (planktonic or biofilm associated), and the impact of organic and inorganic components of the test model on biocidal activity, let alone the sheer multitude of agents and microbial taxa that could form testing combinations^21^.

## Complexity of FPE microbial communities

The success of food safety and risk management programmes in FPEs can be defined by the reduction in the level of microbes in the environment and the disruption of transmission pathways from the environment to foods/ingredients. Despite the significant efforts to reduce microbial loads in FPE, they remain inherently non-sterile. Microbial communities, usually in the form of biofilms, are commonly present^22^. Under selective pressure, these microbial communities will establish and adapt. Multiple sources can continually introduce diverse microbial populations into facilities, including from food ingredients and supplies, transportation infrastructure, workforce, and water and air-handling systems. Common reservoirs where microbes are expected to be present in FPE and that are indicative of the total flora include ‘non-food contact’ surfaces such as floors, drains, ventilation systems and walls. These areas can act as sources of contamination and transmit to food contact surfaces and other connected areas of an FPE^23^. Over time, environmental pressures from an FPE’s site-specific circumstances co-select for multiple microbial strains uniquely adapted to the facility, leading to the establishment of adapted and resistant microbial communities capable of surviving in specific harbouring sites^24–26^.

The interactions within these microbial communities can be diverse and dynamic^27,28^, where microbes may engage in synergistic relationships that enhance their ability to persist under environmental stressors, such as through metabolic cooperation, co-adhesion, or quorum sensing. In many circumstances, complex biofilms evolve that further shield microbial cells from eradication and potentially promote resistance to commonly used biocides^29–32^. We expand on their importance in Box 1. These ecological dynamics have direct implications for hygiene strategy, because the structure and behaviour of microbial communities determine how well interventions perform in practice. Antagonistic relationships can also occur, in which certain microbial species inhibit the growth or survival of others through competitive exclusion, production of antimicrobial compounds, or resource limitation^33,34^. These naturally occurring antagonistic interactions offer promising opportunities for biocontrol strategies, where non-pathogenic or beneficial microbes may be intentionally promoted or introduced to suppress the establishment and transmission of pathogens in FPEs.

As microbial species adapt to these stresses, a more comprehensive understanding of the facility’s total microbiota, along with continuous evaluation of disinfectant efficacy, is essential for developing informed control strategies to mitigate microbial risks and to subsequently monitor and validate that control measures are effectively shifting microbial populations towards a lower level of food safety risk.

Box 1 Biofilm ecologies in the context of Food Production Environments *Biofilms can be the dominant and resilient state of bacteria in food facilities and are one of the reasons a robust culture of food safety is essential, going beyond conventional surface cleaning. This Box draws on Liu* et al*. (2024) as a primary reference, alongside additional literature cited throughout this perspective*.

Biofilms are structured microbial communities in which cells attach to surfaces and become embedded within a protective extracellular matrix. This lifestyle represents a predominant state for many bacteria, enabling persistence under challenging conditions. Compared to free-floating (planktonic) cells, biofilm-associated bacteria display markedly higher tolerance to stress, including disinfectants, antimicrobials, desiccation, and physical cleaning. These traits arise from several mechanisms, such as reduced metabolic activity in deeper layers, limited penetration of disinfectants through the matrix, and coordinated stress responses at the community level^32^.

In food production environments, these features mean that once biofilms establish on equipment, drains, or hard-to-clean niches, they can act as chronic reservoirs. Biofilms support repeated cycles of contamination, allow gene exchange that can spread resistance traits, and often harbour “persister” cells that survive even aggressive cleaning regimes.

For food safety, the implications include:

- **Detection gaps**: routine presence/absence testing may fail to accurately characterise the composition of biofilms that can intermittently release cells into connected areas of the facility.
- **Difficulty of removal**: conventional cleaning and disinfection may not be sufficient unless specifically designed to disrupt biofilm structure.
- **Hygiene first**: maintaining dry conditions, purposeful surface design, and targeted hygiene measures is more effective than attempting to remove mature biofilms once established.

## Current approaches to environmental monitoring and control

On the likelihood that microbial niches will form in FPE, it is a common requirement that food producers implement an environmental monitoring programme to sample and identify the presence or absence of prioritised food safety risks, such as *L. monocytogenes*. Swabs of ‘food contact’ and ‘non-food contact’ surfaces - most commonly from ‘high care/high risk’ areas of a FPE (e.g., those producing ready-to-eat foods, and susceptible to post-cook microbial contamination) - are sent to private or reference testing laboratories for detection of target microbial pathogens. Depending on legislative requirements, industry guidance, or specific supply chain standards (e.g., specifications set by a retailer for their suppliers), if pathogens are detected beyond a certain threshold in food products (e.g., EU law - Regulation (EC) No 2073/2005 - requires less than 100 colony forming units of *L. monocytogenes* per gram of ready-to-eat products that do not support *Listeria* growth^35^), this may trigger further environmental or product monitoring to support root cause analyses and risk assessment. Enhanced cleaning and disinfection measures may also be employed to mitigate immediate risks, particularly where pathogen-containing biofilms are suspected in the FPE^11^.

It is also preferred to prevent biofilms rather than to find biofilms. Trending and analyses of environmental monitoring data provide critical information on areas within a FPE that are vulnerable to the formation of microbial harbourage sites, informing customised control measures and mitigation plans. These new lines of defence can include a series of engineering and infrastructure controls, employee education, supply chain management and rigorous cleaning and disinfection plans that seek to prevent the formation of these niches (Fig. 1). Given that a ‘one-size-fits-all’ approach is often ineffective, multiple tailored strategies are typically required for effective microbial control. In this regard, biofilms have a significant part to play in environmental hygiene management plans, where areas that are less accessible to disinfectants are of particular concern, and each harbourage site may be unique in relation to the degree of microbial attachment, density, microbial composition, metabolic processes, and biocide resistance phenotypes.

In developing this perspective, we draw on insights informally shared by industry stakeholders across roles, including company microbiologists, quality managers, and technical managers - who have provided a viewpoint on the practical challenges, evolving strategies, and embedded knowledge that are the foundation of day-to-day operations to manage microbial risks in FPEs^36,37^.

The food industry holds a clear responsibility to safeguard the public, and robust practices in risk management and due diligence are already embedded across the sector. Within the overarching culture of food safety that shapes behaviours and decisions in the FPE, these stakeholders emphasised four key lines of defence against microbial contamination: preventing the **entry,** **harbourage**, and **spread** of hazards - and when contamination does occur, taking swift action to **kill and remove** pathogens from the environment:

### A. Preventing Entry (*Operations*)

Microbial risks may enter FPEs via raw ingredients, packaging, personnel, or equipment - posing particular concern in high-care or high-risk areas where no subsequent kill step is applied, for example, in post-cook or ready-to-eat food production. Stakeholders emphasised that, in these settings, risk mitigation must begin at the source, with enhanced supplier assurance and quality control measures to reduce bioburden prior to entry. This includes, using an example from the UK, the adoption of the *Microbiological Guidance for Growers (MGG4)* issued by the Chilled Food Association^16^, which outlines standards for raw produce intended for high-care use.

Stakeholders also stressed that food safety knowledge and decision-making capability must extend across all levels of the workforce. Several participants described challenges in maintaining microbiological awareness among regulatory inspectors and auditors, noting that limited expertise in microbial ecology and hygiene may hinder meaningful engagement. A recurring sentiment was that ‘*to understand microbiology means mentorship’* - underscoring the value of hands-on learning and knowledge transfer. To support this, organisations are investing in structured training and competency frameworks that embed microbiological understanding across the workforce - not just within technical teams, but in all roles that influence food safety (e.g. engineers). For many businesses and trade associations, this has meant renewed efforts to create educational materials that ‘*make technical approachable’* so that they can broadly increase awareness of food safety principles across their team, regardless of expertise.

In this view, preventing microbial entry is not only a matter of process and supply chain design, but also of sustained organisational learning. Ensuring all staff understand why specific practices are implemented was described as essential to a robust food safety culture^38^. Confounding this objective, frequent supplier changes, staff turnover, or the introduction of new raw materials can introduce variability and vulnerabilities - highlighting the need for continuous vigilance.

### B. Preventing Harbourage (*Infrastructure*)

The presence of persistent microbial contamination in FPEs is often linked to structural features that enable microbes to establish protected niches^22^. Biofilms commonly develop in hard-to-reach areas - including crevices, porous surfaces, and worn or damaged materials - where effective disinfection is difficult. Intermediate vectors such as wheeled equipment, conveyor belts, cleaning tools, footwear, and airborne droplets generated during washdowns can also mobilise microbes between zones^39^. These pathways contribute to both the transmission and persistence of contamination and must be actively managed as part of a broader infrastructure and hygiene strategy.

Stakeholders emphasised that these risks can be significantly reduced through the application of hygienic design principles during facility construction, refurbishment, or equipment selection. Designs that favour smooth, edge-free, and non-absorbent surfaces help reduce the potential for microbial accumulation and biofilm development^40^. Additionally, an often-overlooked but important control measure is maintaining environmental dryness. Keeping ambient humidity low can disrupt biofilm formation, while even microscopic areas with moisture in otherwise dry facilities may still permit surface colonisation^41–43^.

Facilities with ageing infrastructure or deferred maintenance are identified as vulnerable. Amongst our stakeholders, expansion joints, cracked floor seals, degraded polymer surfaces, and poorly fabricated installations were cited as common harbourage points for *L. monocytogenes*. Engineering and maintenance teams may prioritise mechanical function or throughput over microbiological risk - *‘it is often a surprise to engineers what is actually susceptible to microbial contamination, because their focus is on keeping the factory running, not on microbiology’* - underscoring the importance of targeted food safety training aligned with their operational responsibilities.

To address this, some stakeholders described efforts to better integrate engineering and hygiene goals - for example, through condition monitoring and *‘fix before fail’* maintenance strategies that reduce unplanned downtime while supporting food safety outcomes. Knowledge-sharing through mentorship and role-specific training was again highlighted as essential to embedding hygienic thinking into daily operations.

### C. Preventing spread (*Monitoring*)

Environmental monitoring in FPEs not only detects pathogens but also helps identify individual and connected zones susceptible to microbial accumulation and biofilm formation. Stakeholders highlighted the use of low-complexity tools, such as chromogenic sprays, swabs, and hydrogen peroxide-based detection methods, to visualise potential hotspots^44,45^. In the UK, commercial assays and reagents like FreshCheck®, Biofinder®, and TBF® 300 are in routine use by some food businesses as rapid, colour-based indicators of hygiene status, enabling technical teams to quickly survey areas of concern within a facility. As one stakeholder noted, *‘contamination is invisible - and simplified results that can be understood by everyone’* are key. Culture-based approaches, PCR, and genomics are not always accessible at point-of-use, whereas bright indicator colours offer intuitive feedback, using food-safe chemistry that triggers rapid colour changes with minimal ambiguity and no hardware requirements.

Where concern arises, these zones may be flagged for additional interventions. Targeted treatments with enzymatic or oxidising agents can be applied to disrupt suspected biofilms, followed by re-evaluation to confirm efficacy. Though not substitutes for routine hygiene practices, such strategies are often rotated into cleaning programmes to reinforce control - particularly in areas that are structurally or operationally difficult to clean.

These practices reflect a broader principle echoed by stakeholders: the importance of layered, responsive monitoring and hygiene systems that integrate routine procedures with targeted actions. At the core of this system is the imperative to remove what cannot be prevented - through cleaning and disinfection measures designed to actively reduce microbial presence in the FPE.

### D. Kill and remove (*or ‘Seek and Destroy’*)

Routine C&D programmes are foundational to microbial risk control in FPEs^11^. While disinfection often receives the emphasis, stakeholders stressed that effective cleaning - the physical and chemical removal of organic matter that can shield microbial cells - is the critical first step^46^. While the impact of cleaning is dependent on many factors relating to the soil and substrate and cleaning agent, the act of cleaning alone can remove up to 99.8% of microbes from surfaces^47^, enabling disinfectants to reach their targets and act on biofilms or residual contaminants.

Cleaning strategies are tailored to the type of soil present^48^. Alkaline detergents (e.g., sodium hydroxide, potassium hydroxide) are highly effective for breaking down proteins and fats; acidic agents (e.g., phosphoric or nitric acid) remove scale; and solvent-based cleaners address grease residues. Enzyme-based detergents may also be periodically incorporated to degrade biofilm matrices and improve the efficacy of downstream disinfection. Mechanical energy - such as brushing, scraping, scrubbing, or low-pressure washing - enhances removal, though high-pressure systems must be used cautiously, as they can aerosolise pathogens and increase the risk of cross-contamination^39^.

Following cleaning, disinfectants are selected based on microbial spectrum, surface compatibility, and regulatory status. Common agents include quaternary ammonium compounds (QACs), chlorine-based disinfectants, hydrogen peroxide, alcohols, peracetic acid, and iodophors^49–52^. In the UK and EU, such products are regulated under the Biocidal Products Regulation (BPR), with oversight from the Health and Safety Executive (HSE) and the European Chemicals Agency (ECHA). Under the EU BPR (Regulation (EU) No 528/2012), disinfectants must demonstrate efficacy against relevant microorganisms using standardised European test methods (for example EN-series surface and suspension tests) before approval for use. These regulatory standards set baseline expectations for performance, but as discussed here, additional facility-specific validation is often required in complex food production environments. Disinfectant performance is influenced by a range of factors - including surface condition, biocide concentration, contact time, and the presence of organic material^39,53^. While QACs offer broad-spectrum coverage, they are less effective against spores and mature biofilms^54^, and the evolution of reduced susceptibility can occur^55^. In contrast, oxidising agents such as peracetic acid and hydrogen peroxide are often favoured in enclosed systems for their efficacy against structured communities^56^.

There is growing interest in emerging agents such as hypochlorous acid (HOCl), which combines broad-spectrum efficacy with low toxicity, biodegradability, and a relatively mild environmental profile^57^. These products are increasingly being adopted to support sustainable hygiene strategies while reducing reliance on harsher chemistries.

Food businesses may ask: *‘Will traditional chemistry be enough to control this risk? If not, then seek additional solutions.’* Even the most broadly acting biocides can be constrained by context, and if the *‘chemistry’* is insufficient, food businesses may adopt incremental control strategies. Enzyme-based cleaners can be used to delayer biofilms, increasing microbial susceptibility to subsequent disinfection^58^. Other enhancements - including antimicrobial surface materials, UV air treatment, HEPA filtration, ozone, and time-temperature-controlled delivery systems - can be integrated to supplement conventional C&D. These are sometimes deployed on a rotating basis or as part of capital investment projects, particularly where microbial harbourage or airflow-driven contamination is persistent.

Surface topography, facility layout, and environmental variables (e.g., dew point, condensation risk) can all affect outcomes. Regimes must therefore be tailored to the specific realities of each facility. The goal of hygiene programmes is to *‘influence as much as you can’* - through layered, fit-for-purpose controls that reflect an ongoing commitment to food safety and continuous improvement.

## New solutions and opportunities

Innovation in microbial control is not driven by novelty alone - it reflects a culture of food safety grounded in evidence-based action, economic feasibility, and shared learning. While the fundamentals of hygiene remain constant, research networks and forward-looking food businesses are collaborating to address persistent research needs and to close gaps in detection, interpretation, and intervention.

### A. Pursuit of innovation

Food safety is widely viewed as a shared priority, not a source of competitive advantage. Increasingly, progressive businesses with a culture of open innovation are joining collaborative networks such as the Food Safety Research Network^59^ that connect microbiologists, material scientists, engineers, and regulators with the personnel responsible for hygiene on the factory floor. These partnerships are grounded in practical questions reflective of a ‘*healthy paranoia’* to identify hard-to-reach risks: *‘What exactly are we trying to clean off? Where are the microbes in the system? What survives cleaning, and how does the microbiome re-establish itself? How do organisms transit across equipment, people, and processes?* And critically - *which microbes pose the greatest risk to consumer safety?’*

These questions can’t be answered in isolation. For businesses attuned to microbial risk - often through their deep product knowledge - there is a desire for business-to-business learning. What insights can be shared across facilities or sectors to better understand where risks reside and how microbial populations respond to selective pressures? Solutions require critical thinking and support to evaluate new technologies, interpret complex results, and translate data into meaningful action - all within the real-world constraints of time, cost, and other operational workloads. We discuss here innovations and collaborative approaches that are providing solutions.

### B. Preventing harbourage: surfaces and coatings

Preventing microbial establishment at the source is a critical component of effective hygiene practice. While traditional disinfectants focus on reducing microbial loads after contamination has occurred, newer solutions aim to block initial attachment and disrupt biofilm maturation before it begins.

Surface engineering plays a central role. Hygienic design, characterised by smooth, edge-free construction, chemically resistant materials, and robust sealing compounds, continues to advance in tandem with processing technologies. Alongside this, passive antimicrobial surfaces offer a supplementary layer of control, especially in areas that are difficult to access or clean thoroughly^60,61^.

These include coatings embedded with silver or copper ions, known for their broad-spectrum antimicrobial activity^62^; photocatalytic surfaces that generate reactive oxygen species under light exposure^63^; and polymer-based films infused with quaternary ammonium compounds or organic acids^64^. Some approaches rely on contact-kill mechanisms, while others release antimicrobials gradually over time.

Clean-in-place (CIP) systems represent another major investment in hygiene infrastructure - automating the cleaning of pipes, tanks, and closed-loop systems without disassembly. When effectively designed and validated, CIP systems can significantly reduce harbourage risk in high-throughput, hard-to-access, or fluid-handling environments^39,65^.

However, cost remains a major barrier to widespread adoption. ‘*The passive approach is a great way forward*,’ one stakeholder noted, ‘*but the costs to implement are the problem*.’ Even so, several businesses are beginning to incorporate surface innovations and CIP upgrades into infrastructure renewal - prioritising areas with known harbourage risk, and weighing hygiene gains against the long-term returns on investment.

### C. Preventing spread: genomics and metagenomics as a guidepost

Understanding the fuller microbial ecology of a facility, rather than targeting only specific pathogens, is emerging as a powerful lens for action. Metagenomics, particularly shotgun sequencing combined with whole-genome sequencing of recovered taxa, is now being increasingly explored to detect microbial shifts associated with contamination, spoilage, or the application of disinfectants^66–68^.

These approaches provide both taxonomic and functional insight, helping teams highlight persistently present taxa across a facility or a targeted process area and support investigations into potential sources and evaluate whether control measures are truly reshaping microbial populations. As reviewed by Mather et al., when combined with business-specific expertise and knowledge of existing hygiene systems, genomics and metagenomics offer complementary data to refine risk assessments and inform hygiene strategies that align with HACCP frameworks, enabling more precise interventions^69^.

Several stakeholders noted that metagenomics could help them *‘close the net’* on unknown risks by identifying gaps in understanding of microbial communities that may signal emerging issues - though always at a cost to perform. At present, shotgun metagenomics remains largely research-led in FPEs; in most cases, lower-cost 16S rRNA or ITS surveys are used instead to profile microbial communities, though these mainly provide taxonomic data and lack the functional insights of shotgun sequencing. Sampling in under-monitored zones requires planning, operator coordination, and financial investment. While not intended for routine use, metagenomics can play a critical calibrating role: establishing a broader baseline of a facility’s microbiota and informing the development of targeted follow-on assays for key taxa of concern.

While metagenomics offers powerful insights into microbial community structure, many food businesses are already finding immediate value in sequencing individual isolates from their facilities. Whole-genome sequencing of isolates can distinguish persistent from transient strains, trace their spread within facilities, and highlight potential virulence or resistance determinants^69^. In practice, some businesses report that this resolution is a critical problem-solving tool, enabling targeted hygiene interventions or process redesign in ways that routine presence/absence testing or even broader metagenomic profiles cannot. For businesses, the value of these tools lies in balancing simple, actionable outputs with the richer data needed for root-cause analysis and long-term control.

At this stage, these methods remain highly specialised and are best pursued in partnership with research institutes and funders committed to long-term capability-building. Stakeholders have shared that metagenomics can ‘*help verify what changes after a new control’*, offering a before-and-after view of microbial communities that would otherwise remain invisible to businesses. Used judiciously, it is a tool to better understand microbial reservoirs and inform management decisions - especially when safety issues or spoilage persist under the lens of routine tools.

### D. Killing with confidence: standardising disinfectant validation

Despite their central role in microbial control, the tools used to validate disinfectant efficacy remain poorly aligned with the complexity of FPEs^19,70^. Standardised laboratory tests - for example, EN 1276, Minimum Inhibitory Concentration (MIC), or Minimum Bactericidal Concentration (MBC) assays assess planktonic cultures of reference strains, tested in liquid suspensions or on uniform surfaces in well-controlled experimental settings^71^. While these protocols are foundational for regulatory approval and comparability, they often fail to reflect real-world performance.

Small changes in parameters - contact time, temperature, soil load, growth phase - can dramatically alter outcomes^72^. For example, published MIC values for *Enterococcus faecalis* ATCC 29212 against chlorhexidine have ranged from <1 mg/L to over 25 mg/L depending on method and conditions^73^. Biofilm-associated cells, common in FPEs, often require biocide concentrations many times higher than those effective against planktonic populations^74–76^. Yet most lab assays remain anchored in reductionist models that overlook these ecological realities.

Even where biofilm-specific metrics exist - such as the minimal biofilm eradication concentration (MBEC), biofilm prevention concentration (BPC), or biofilm bactericidal concentration (BBC) - there is no consensus on standard definitions, test systems, or regulatory benchmarks^77–79^. And when commercial disinfectants, often formulated with multiple active substances and surfactants, are rarely assessed in their full-product formulations, a disconnect between test results and operational confidence can be created. Without better predictive data, some businesses may feel compelled to either ‘over disinfect’ (at cost) or accept uncertainty. It is preferred to know ‘*what works, and where.”*

In response, food businesses can supplement routine testing with research-driven challenge trials - using facility-relevant soils, resident microbial strains, or bespoke biofilm models to evaluate how products actually perform under local conditions. These approaches will allow technical teams to verify claims, calibrate expectations, and build hygiene strategies that reflect their true operating environment.

At the regulatory level, initiatives such as the EU COST Action *RegulatoryToolBox* (CA23152) are working to harmonise test criteria and enable cross-sector consensus on what constitutes meaningful disinfectant efficacy^80^. True validation cannot rely on universal tests alone; instead, site-specific factors, including surface materials, layout, production cycles, climate, and microbial ecology (e.g., representative strains), can each play a decisive role in disinfectant performance^48,81^. This evolving framework acknowledges that some detections are expected, not all reductions signify success, and context is everything. A single hygiene failure may not reflect systemic breakdown, while the absence of findings - if supported by robust, site-tailored verification - can build confidence. As we ‘*learn to value null results*’ one stakeholder advised that ‘*they may be telling you your system is working*.’

The challenge is not only to balance method harmonisation with field customisation, but also to include non-microbiological measures of success. Verification must extend to product impact - with indicators such as reduced recalls, fewer customer complaints, or lower rates of reported illness. If interventions do not translate into measurable product or consumer benefit, then their rationale should be re-evaluated. In this context, effective microbial control becomes not just a regulatory obligation, but a strategic decision rooted in evidence of the real-world outcomes that matter most to businesses and consumers.

### E. Modelling reality: multi-species and biofilm assays

Considering the essential context of FPE microbes in biofilms, it’s increasingly clear that the gold standard for disinfectant testing must evolve - away from single strains on polished steel or new plastics, and toward models that better mimic the ecology and wear-and-tear of real factory surfaces^53^.

A significant proportion of laboratory biofilm studies still rely on single strains grown on idealised surfaces, in defined media and fixed conditions^60^. These models fail to capture the cooperative and competitive interactions that emerge within complex microbial consortia. In multispecies biofilms, certain taxa may produce extracellular polymeric substances (EPS), detoxifying enzymes, or signalling compounds that induce stress responses in neighbouring cells^82–84^. This interspecies interaction can result in markedly higher tolerance to biocides - especially in biofilms containing families such as *Pseudomonadaceae* and *Xanthomonadaceae*^85,86^. Such findings increasingly challenge the relevance of mono-species assays as benchmarks for cleaning efficacy in industrial settings.

In response, the development of multi-species biofilm models has gained momentum, driven by the need for more realistic and predictive test systems. When integrated with omics approaches, such as metagenomics, transcriptomics, and metabolomics, these models provide powerful insights into quorum sensing, metabolic complementation, communal stress responses, and microbial succession. They are now being used not only to evaluate disinfectant performance, but also to test new materials, validate cleaning protocols, and understand how microbial communities adapt over time. Importantly, however, biocide susceptibility is rarely measured longitudinally, meaning we have little understanding of whether, and how, microbial populations in food production environments are evolving increased tolerance.

This gap underscores the need for analytical tools that can track biofilm resilience and recovery more directly. New approaches such as microcalorimetry (e.g., CalScreener™) and flow-cell systems enable researchers and technical teams to monitor metabolic recovery, structural integrity, and regrowth potential following treatment^87^. While standardisation remains a challenge, given the wide variability in species combinations, nutrient conditions, surface materials, and methodologies to measure biofilms, we are now moving toward assays that capture resilience and ecological behaviour, not just survival endpoints.

As one stakeholder put it: *‘Not all cleaning success is visible - we need methods that tell us more than just ‘did we kill something in a petri dish?”* The next generation of biofilm assays must reflect not just the presence or absence of a target organism, but the broader ecological behaviours that underpin microbial survival. By building models that better simulate the physical and biological complexity of factory settings, we stand to improve both the realism and reliability of hygiene verification in the food industry.

## Conclusion

Drawing from stakeholder insight and current microbiological research, we outline the following recommendations for innovation-driven food safety activities, where food businesses and research partners are jointly working to future-proof hygiene and microbial control strategies in food production environments:

### A. Innovation and collaboration in a culture of food safety

- Invest in long-term partnerships between food businesses and research institutions to co-develop practical tools that address real-world hygiene challenges, not just laboratory endpoints.
- Leverage internal investment alongside public research programmes to support microbial ecology studies and build capabilities in risk interpretation and hygiene innovation.
- Assess practicality and trade-offs (e.g., cost, regulation, process compatibility) early in the development cycle to reduce barriers to adoption.

### B. Advances to prevent and monitor microbial presence and transmission

- Evaluate alternative antimicrobials and materials - such as bacteriophages, enzymatic treatments, antimicrobial polymers, and photocatalytic surfaces - based on their context-specific effectiveness, safety profile, and ecological impact.
- Integrate rapid detection tools (e.g., chromogenic sprays) into existing hygiene verification systems to guide timely, localised interventions.
- Use genomics and/or metagenomics to establish a microbial baseline of local biota to detect meaningful shifts in community composition associated with spoilage or safety risks, including the presence of persisting strains.

### C. Modelling and validation of disinfectants to kill and remove evolved biofilms

- Benchmark in-use disinfectants against facility-relevant MIC/MBC thresholds using resident strains and representative surface types.
- Supplement standardised assays with site-specific challenge models to validate real-world efficacy and inform hygiene programme adjustments.
- Interpret results probabilistically and longitudinally - not all positives signal failure, and not all reductions guarantee success.
- Adopt multi-species biofilm models - particularly for validating interventions in hard-to-clean zones - and integrate them with omics tools to reveal community-level responses.
- Incorporate additional product and process quality indicators (e.g., customer complaints), not just CFU counts, to evaluate the longer-term impact of hygiene measures and residual risk.

## Data Availability

No datasets were generated or analysed during the current study.

## References

[1] Møretrø, T. & Langsrud, S. Residential bacteria on surfaces in the food industry and their implications for food safety and quality. *Compr. Rev. Food Sci. Food Saf.* **16**, 1022–1041 (2017). 33371605 10.1111/1541-4337.12283

[2] UK Health Security Agency. Shiga toxin-producing *Escherichia coli* (STEC) data: 2024. https://www.gov.uk/government/publications/escherichia-coli-e-coli-o157-annual-totals/shiga-toxin-producing-escherichia-coli-stec-data-2024.

[3] UK Health Security Agency. *Listeriosis in England and Wales: Summary for* 2024. https://www.gov.uk/government/publications/listeria-monocytogenes-surveillance-reports/listeriosis-in-england-and-wales-summary-for-2024 (2024).

[4] UK Health Security Agency. *Non-Typhoidal**Salmonella Data 2015 to* 2024. https://www.gov.uk/government/publications/salmonella-national-laboratory-and-outbreak-data/non-typhoidal-salmonella-data-2015-to-2024.

[5] UK Health Security Agency. *Campylobacter Data 2015 to* 2024. https://www.gov.uk/government/publications/campylobacter-infection-annual-data/campylobacter-data-2015-to-2024.

[6] Global Food Safety Initiative. *A Culture of Food Safety - A Position Paper from the Global Food Safety Initiative (GFSI**)*. https://mygfsi.com/wp-content/uploads/2019/09/GFSI-Food-Safety-Culture-Full.pdf (2018).

[7] Pai, A. S., Jaiswal, S. & Jaiswal, A. K. A comprehensive review of food safety culture in the food industry: leadership, organizational commitment, and multicultural dynamics. *Foods* **13**, 4078 (2024). 39767017 10.3390/foods13244078 PMC11675119

[8] Chilled Food Association. *Principles of an Environmental Monitoring Program for the Management of* Listeria monocytogenes. https://www.chilledfood.org/wp-content/uploads/2023/07/Principles-of-an-environmental-monitoring-program-for-L-monocytogenes-v1-10-7-23.pdf (2023).

[9] Herrera, A. G. The Hazard Analysis and Critical Control Point System in Food Safety. In *Public Health Microbiology* vol. 268 235–280, Humana Press, New Jersey, (2004). 10.1385/1-59259-766-1:235 15156035

[10] Hulebak, K. L. & Schlosser, W. Hazard Analysis and Critical Control Point (HACCP) History and Conceptual Overview. *Risk Anal.* **22**, 547–552 (2002). 12088233 10.1111/0272-4332.00038

[11] Malley, T. J. V., Butts, J. & Wiedmann, M. Seek and destroy process: Listeria monocytogenes process controls in the ready-to-eat meat and poultry industry. *J. Food Prot.* **78**, 436–445 (2015). 25710164 10.4315/0362-028X.JFP-13-507

[12] Agüeria, D. A., Libonatti, C. & Civit, D. Cleaning and disinfection programmes in food establishments: a literature review on verification procedures. *J. Appl. Microbiol.* **131**, 23–35 (2021). 33300256 10.1111/jam.14962

[13] Rathore, A. S. & Kapoor, G. Implementation of quality by design toward processing of food products. *Prep. Biochem. Biotechnol.* **47**, 435–440 (2017). 28402220 10.1080/10826068.2017.1315601

[14] Pakdel, M., Olsen, A. & Bar, E. M. S. A Review of Food Contaminants and Their Pathways Within Food Processing Facilities Using Open Food Processing Equipment. *J. Food Prot.* **86**, 100184 (2023). 37865163 10.1016/j.jfp.2023.100184

[15] BRCGS. *Global Standard Food Safety*. https://www.brcgs.com/product/global-standard-food-safety-(issue-9)/p-13279/ (2022).

[16] Chilled Food Association. *Microbiological Guidance for Produce Suppliers to Chilled Food Manufacturers*. https://www.chilledfood.org/cfa-updates-and-revises-its-internationally-influential-microbiological-guidance-for-growers-mgg4/ (2025).

[17] Helliwell, R. et al. Rethinking the words hotspot reservoir and pristine in the environmental dimensions of antimicrobial resistance. *Npj Antimicrob. Resist.* **3**, 11 (2025). 39984758 10.1038/s44259-025-00080-9 PMC11845593

[18] Maillard, J.-Y. Impact of benzalkonium chloride, benzethonium chloride and chloroxylenol on bacterial antimicrobial resistance. *J. Appl. Microbiol.* **133**, 3322–3346 (2022). 35882500 10.1111/jam.15739 PMC9826383

[19] Wesgate, R., Grasha, P. & Maillard, J.-Y. Use of a predictive protocol to measure the antimicrobial resistance risks associated with biocidal product usage. *Am. J. Infect. Control* **44**, 458–464 (2016). 26810885 10.1016/j.ajic.2015.11.009

[20] Bland, R., Brown, S. R. B., Waite-Cusic, J. & Kovacevic, J. Probing antimicrobial resistance and sanitizer tolerance themes and their implications for the food industry through the *Listeria monocytogenes* lens. *Compr. Rev. Food Sci. Food Saf.* **21**, 1777–1802 (2022). 35212132 10.1111/1541-4337.12910

[21] O’Reilly, P., Loiselle, G., Darragh, R., Slipski, C. & Bay, D. C. Reviewing the complexities of bacterial biocide susceptibility and in vitro biocide adaptation methodologies. *Npj Antimicrob. Resist.* **3**, 39 (2025). 40360746 10.1038/s44259-025-00108-0 PMC12075810

[22] Carpentier, B. & Cerf, O. Review — Persistence of Listeria monocytogenes in food industry equipment and premises. *Int. J. Food Microbiol.* **145**, 1–8 (2011). 21276634 10.1016/j.ijfoodmicro.2011.01.005

[23] Chmielewski, R. A. N. & Frank, J. F. Biofilm formation and control in food processing facilities. *Compr. Rev. Food Sci. Food Saf.* **2**, 22–32 (2003). 33451238 10.1111/j.1541-4337.2003.tb00012.x

[24] Cobo-Díaz, J. F. et al. Microbial colonization and resistome dynamics in food processing environments of a newly opened pork cutting industry during 1.5 years of activity. *Microbiome* **9**, 204 (2021). 34645520 10.1186/s40168-021-01131-9 PMC8515711

[25] Tompkin, R. B. Control of Listeria monocytogenes in the Food-Processing Environment. *J. Food Prot.* **65**, 709–725 (2002). 11952224 10.4315/0362-028x-65.4.709

[26] Liu, X., Yao, H., Zhao, X. & Ge, C. Biofilm formation and control of foodborne pathogenic bacteria. *Molecules* **28**, 2432 (2023). 36985403 10.3390/molecules28062432 PMC10058477

[27] Li, Q. et al. Formation of multispecies biofilms and their resistance to disinfectants in food processing environments: a review. *J. Food Prot.* **84**, 2071–2083 (2021). 34324690 10.4315/JFP-21-071

[28] Fagerlund, A., Langsrud, S. & Møretrø, T. Microbial diversity and ecology of biofilms in food industry environments associated with Listeria monocytogenes persistence. Curr. *Opin. Food Sci.* **37**, 171–178 (2021).

[29] Rodríguez-López, P., Rodríguez-Herrera, J. J., Vázquez-Sánchez, D. & López Cabo, M. Current knowledge on Listeria monocytogenes biofilms in food-related environments: incidence, resistance to biocides, ecology and biocontrol. *Foods* **7**, 85 (2018). 29874801 10.3390/foods7060085 PMC6025129

[30] Alvarez-Ordóñez, A., Coughlan, L. M., Briandet, R. & Cotter, P. D. Biofilms in Food Processing Environments: Challenges and Opportunities. Annu. *Rev. Food Sci. Technol.* **10**, 173–195 (2019). 10.1146/annurev-food-032818-121805 30653351

[31] Voloshchuk, O. et al. Pseudomonadaceae increased the tolerance of Listeria monocytogenes to sanitizers in multi-species biofilms. *Food Microbiol*. **128**, 104687 (2025). 39952739 10.1016/j.fm.2024.104687

[32] Liu, H. Y., Prentice, E. L. & Webber, M. A. Mechanisms of antimicrobial resistance in biofilms. *Npj Antimicrob. Resist.* **2**, 27 (2024). 39364333 10.1038/s44259-024-00046-3 PMC11445061

[33] Giaouris, E. et al. Intra- and inter-species interactions within biofilms of important foodborne bacterial pathogens. Front. Microbiol. 6, (2015). 10.3389/fmicb.2015.00841 PMC4542319 26347727

[34] Zhao, T. et al. Control of *Listeria* spp. by Competitive-Exclusion Bacteria in Floor Drains of a Poultry Processing Plant. *Appl. Environ. Microbiol.* **72**, 3314–3320 (2006). 16672472 10.1128/AEM.72.5.3314-3320.2006 PMC1472388

[35] European Commission. *Commission Regulation (EC) No 2073/2005*. https://www.legislation.gov.uk/eur/2005/2073/contents (2005).

[36] National Biofilms Innovation Centre. *Biofilm Methodologies and Food Sector Regulation*. (University of Southampton, 2025). 10.5258/BIOFILMS/016.

[37] Gilmour, M. et al. *Advancing Shiga Toxin-Producing* E. coli *(STEC) Diagnostics - Workshop Report*. 10.5281/ZENODO.16921087 (2025).

[38] Griffith, C. J. Food safety: where from and where to? Br. *Food J.* **108**, 6–15 (2006).

[39] Gibson, H., Taylor, J. H., Hall, K. E. & Holah, J. T. Effectiveness of cleaning techniques used in the food industry in terms of the removal of bacterial biofilms. *J. Appl. Microbiol.* **87**, 41–48 (1999). 10432586 10.1046/j.1365-2672.1999.00790.x

[40] European Hygienic Engineering & Design Group. *GL8 Hygienic Design Principles*. (2018).

[41] Ban-Cucerzan, A. et al. PersistenT Threats: A Comprehensive Review Of Biofilm Formation, Control, And Economic Implications In Food Processing Environments. *Microorganisms* **13**, 1805 (2025). 40871309 10.3390/microorganisms13081805 PMC12388178

[42] Chen, L., Rana, Y. S., Heldman, D. R. & Snyder, A. B. Environment, food residue, and dry cleaning tool all influence the removal of food powders and allergenic residues from stainless steel surfaces. *Innov. Food Sci. Emerg. Technol.* **75**, 102877 (2022).

[43] Alonso, V. P. P. et al. Dry surface biofilms in the food processing industry: An overview on surface characteristics, adhesion and biofilm formation, detection of biofilms, and dry sanitization methods. *Compr. Rev. Food Sci. Food Saf.* **22**, 688–713 (2023). 36464983 10.1111/1541-4337.13089

[44] González-Rivas, F., Ripolles-Avila, C., Fontecha-Umaña, F., Ríos-Castillo, A. G. & Rodríguez-Jerez, J. J. Biofilms in the Spotlight: Detection, Quantification, And Removal Methods. *Compr. Rev. Food Sci. Food Saf.* **17**, 1261–1276 (2018). 33350156 10.1111/1541-4337.12378

[45] Lorenzo, F., Sanz-Puig, M., Bertó, R. & Orihuel, E. Assessment Of Performance Of Two Rapid Methods For On-site Control Of Microbial And Biofilm Contamination. *Appl. Sci.* **10**, 744 (2020).

[46] Carpentier, B. & Cerf, O. Biofilms and their consequences, with particular reference to hygiene in the food industry. *J. Appl. Bacteriol.* **75**, 499–511 (1993). 8294303 10.1111/j.1365-2672.1993.tb01587.x

[47] Dunsmore, D. G., Twomey, A., Whittlestone, W. G. & Morgan, H. W. Design And Performance Of Systems For Cleaning Product-contact Surfaces Of Food Equipment: A Review. *J. Food Prot.* **44**, 220–240 (1981). 30836495 10.4315/0362-028X-44.3.220

[48] Maillard, J.-Y. & Pascoe, M. Disinfectants and antiseptics: mechanisms of action and resistance. *Nat. Rev. Microbiol.* **22**, 4–17 (2024). 37648789 10.1038/s41579-023-00958-3

[49] Duze, S. T., Marimani, M. & Patel, M. Tolerance of Listeria monocytogenes to biocides used in food processing environments. *Food Microbiol*. **97**, 103758 (2021). 33653529 10.1016/j.fm.2021.103758

[50] Gallandat, K., Kolus, R. C., Julian, T. R. & Lantagne, D. S. A systematic review of chlorine-based surface disinfection efficacy to inform recommendations for low-resource outbreak settings. *Am. J. Infect. Control* **49**, 90–103 (2021). 32442652 10.1016/j.ajic.2020.05.014 PMC7236738

[51] Castro, D., Ferreri, I., Carvalho, I. & Henriques, M. Long-lasting multi-surface disinfectant: Evaluation of efficiency and durability. *Results Eng.* **16**, 100649 (2022).

[52] Oh, E. et al. Enhanced biocidal efficacy of alcohol based disinfectants with salt additives. *Sci. Rep.* **15**, 3950 (2025). 39890978 10.1038/s41598-025-87811-0 PMC11785731

[53] Hamilton, A. N. et al. A Systematic Review and Meta-Analysis of Chemical Sanitizer Efficacy Against Biofilms of Listeria monocytogenes, Salmonella enterica, and STEC on Food Processing Surfaces. *J. Food Prot.* **88**, 100495 (2025). 40122344 10.1016/j.jfp.2025.100495

[54] Arnold, W. A. et al. Quaternary AmmonIum Compounds: A Chemical Class Of Emerging Concern. *Environ. Sci. Technol.* **57**, 7645–7665 (2023). 37157132 10.1021/acs.est.2c08244 PMC10210541

[55] Martínez-Suárez, J. V., Ortiz, S. & López-Alonso, V. Potential Impact of the Resistance to Quaternary Ammonium Disinfectants on the Persistence of Listeria monocytogenes in Food Processing Environments. *Front. Microbiol.* **2**, 638 (2016). 10.3389/fmicb.2016.00638 PMC4852299 27199964

[56] Abdelshafy, A. M., Neetoo, H. & Al-Asmari, F. Antimicrobial activity of hydrogen peroxide for application in food safety and COVID-19 mitigation: an updated review. *J. Food Prot.* **87**, 100306 (2024). 38796115 10.1016/j.jfp.2024.100306

[57] Block, M. S. & Rowan, B. G. Hypochlorous acid: a review. *J. Oral. Maxillofac. Surg.* **78**, 1461–1466 (2020). 32653307 10.1016/j.joms.2020.06.029 PMC7315945

[58] Dawan, J., Zhang, S. & Ahn, J. Recent advances in biofilm control technologies for the food industry. *Antibiotics* **14**, 254 (2025). 40149064 10.3390/antibiotics14030254 PMC11939704

[59] Food Safety Research Network. *Food Safety Research Network* https://fsrn.quadram.ac.uk/about-the-network/.

[60] Redfern, J., Cunliffe, A. J., Goeres, D. M., Azevedo, N. F. & Verran, J. Critical analysis of methods to determine growth, control and analysis of biofilms for potential non-submerged antibiofilm surfaces and coatings. *Biofilm* **7**, 100187 (2024). 38481762 10.1016/j.bioflm.2024.100187 PMC10933470

[61] Alves, V. F. et al. Hidden places for foodborne bacterial pathogens and novel approaches to control biofilms in the meat industry. *Foods* **13**, 3994 (2024). 39766937 10.3390/foods13243994 PMC11675819

[62] Silvestry-Rodriguez, N., Bright, K. R., Slack, D. C., Uhlmann, D. R. & Gerba, C. P. Silver as a residual disinfectant to prevent biofilm formation in water distribution systems. *Appl. Environ. Microbiol.* **74**, 1639–1641 (2008). 18192431 10.1128/AEM.02237-07 PMC2258619

[63] Ubaldi, F., Valeriani, F., Volpini, V., Lofrano, G. & Romano Spica, V. Antimicrobial activity of photocatalytic coatings on surfaces: a systematic review and meta-analysis. *Coatings* **14**, 92 (2024).

[64] Artusio, F. et al. Broad-spectrum supramolecularly reloadable antimicrobial coatings. *ACS Appl. Mater. Interfaces* **16**, 29867–29875 (2024). 38825754 10.1021/acsami.4c04705 PMC11181266

[65] Pant, K. J., Cotter, P. D., Wilkinson, M. G. & Sheehan, J. J. Towards sustainable Cleaning-in-Place (CIP) in dairy processing: Exploring enzyme-based approaches to cleaning in the Cheese industry. *Compr. Rev. Food Sci. Food Saf.* **22**, 3602–3619 (2023). 37458296 10.1111/1541-4337.13206

[66] Reiche, T. et al. Shifts in surface microbiota after cleaning and disinfection in broiler processing plants: incomplete biofilm eradication revealed by robotic high-throughput screening. *Appl. Environ. Microbiol.* **91**, e02401–e02424 (2025). 40008875 10.1128/aem.02401-24 PMC11921370

[67] Diaz, M. et al. Microbial composition and dynamics in environmental samples from a ready-to-eat food production facility with a long-term colonization of Listeria monocytogenes. *Food Microbiol.* **125**, 104649 (2025). 39448159 10.1016/j.fm.2024.104649

[68] Rolon, M. L. et al. Impact of improved sanitation standard operating procedures on microbial populations at three tree fruit packing facilities. *J. Food Prot.* **88**, 100436 (2025). 39701447 10.1016/j.jfp.2024.100436

[69] Mather, A. E., Gilmour, M. W., Reid, S. W. J. & French, N. P. Foodborne bacterial pathogens: genome-based approaches for enduring and emerging threats in a complex and changing world. *Nat. Rev. Microbiol.* **22**, 543–555 (2024). 38789668 10.1038/s41579-024-01051-z

[70] Holah, J. T. CEN/TC 216: its role in producing current and future European disinfectant testing standards. *Int. Biodeterior. Biodegrad.* **51**, 239–243 (2003).

[71] Bolten, A., Schmidt, V. & Steinhauer, K. Use of the European standardization framework established by CEN/TC 216 for effective disinfection strategies in human medicine, veterinary medicine, food hygiene, industry, and domestic and institutional use – a review. *GMS Hyg. Infect. Control* **17**, (2022). 10.3205/dgkh000417 PMC9487781 36157383

[72] Russell, A. Biocide use and antibiotic resistance: the relevance of laboratory findings to clinical and environmental situations. *Lancet Infect. Dis.* **3**, 794–803 (2003). 14652205 10.1016/s1473-3099(03)00833-8

[73] Pereira, A. P., Antunes, P., Peixe, L., Freitas, A. R. & Novais, C. Current insights into the effects of cationic biocides exposure on Enterococcus spp. *Front. Microbiol.* **15**, 1392018 (2024). 39006755 10.3389/fmicb.2024.1392018 PMC11242571

[74] Maillard, J.-Y. & Centeleghe, I. How biofilm changes our understanding of cleaning and disinfection. *Antimicrob. Resist. Infect. Control* **12**, 95 (2023). 37679831 10.1186/s13756-023-01290-4 PMC10483709

[75] Bridier, A., Briandet, R., Thomas, V. & Dubois-Brissonnet, F. Resistance of bacterial biofilms to disinfectants: a review. *Biofouling* **27**, 1017–1032 (2011). 22011093 10.1080/08927014.2011.626899

[76] Hall-Stoodley, L., Costerton, J. W. & Stoodley, P. Bacterial biofilms: from the Natural environment to infectious diseases. *Nat. Rev. Microbiol.* **2**, 95–108 (2004). 15040259 10.1038/nrmicro821

[77] Thieme, L. et al. MBEC Versus MBIC: the lack of differentiation between biofilm reducing and inhibitory effects as a current problem in biofilm methodology. *Biol. Proced. Online* **21**, 18 (2019). 31528123 10.1186/s12575-019-0106-0 PMC6743098

[78] Coenye, T. & Nelis, H. J. In vitro and in vivo model systems to study microbial biofilm formation. *J. Microbiol. Methods* **83**, 89–105 (2010). 20816706 10.1016/j.mimet.2010.08.018

[79] Kampf, G. Suitability of methods to determine resistance to biocidal active substances and disinfectants—a systematic review. *Hygiene* **2**, 109–119 (2022).

[80] Action CA23152 - Building Consensus on Biofilm Regulatory Decision Making. *COST (European Cooperation in Science & Technology*) https://www.cost.eu/actions/CA23152.

[81] Holah, J. T. Cleaning and disinfection practices in food processing. in *Hygiene in Food Processing* 259–304 (Elsevier). 10.1533/9780857098634.3.259 (2024).

[82] Wicaksono, W. A. et al. Enhanced survival of multi-species biofilms under stress is promoted by low-abundant but antimicrobial-resistant keystone species. *J. Hazard. Mater.* **422**, 126836 (2022). 34403940 10.1016/j.jhazmat.2021.126836

[83] Sanchez-Vizuete, P., Orgaz, B., Aymerich, S., Le Coq, D. & Briandet, R. Pathogens protection against the action of disinfectants in multispecies biofilms. *Front. Microbiol*. **6**, (2015). 10.3389/fmicb.2015.00705 PMC4500986 26236291

[84] Schwering, M., Song, J., Louie, M., Turner, R. J. & Ceri, H. Multi-species biofilms defined from drinking water microorganisms provide increased protection against chlorine disinfection. *Biofouling* **29**, 917–928 (2013). 23879183 10.1080/08927014.2013.816298

[85] Pang, X. Y., Yang, Y. S. & Yuk, H. G. Biofilm formation and disinfectant resistance of *Salmonella* sp. in mono- and dual-species with *Pseudomonas aeruginosa*. *J. Appl. Microbiol.* **123**, 651–660 (2017). 28644912 10.1111/jam.13521

[86] Rolon, M. L., Voloshchuk, O., Bartlett, K. V., LaBorde, L. F. & Kovac, J. Multi-species biofilms of environmental microbiota isolated from fruit packing facilities promoted tolerance of Listeria monocytogenes to benzalkonium chloride. *Biofilm* **7**, 100177 (2024). 38304489 10.1016/j.bioflm.2024.100177 PMC10832383

[87] Morazzoni, C. et al. Proof of concept: real-time viability and metabolic profiling of probiotics with isothermal microcalorimetry. *Front. Microbiol.* **15**, 1391688 (2024). 38962141 10.3389/fmicb.2024.1391688 PMC11220157

